# Outcomes of Various Interventions for First-Time Perianal Abscesses in Children

**DOI:** 10.1155/2016/9712854

**Published:** 2016-01-05

**Authors:** Alexander Juth Karlsson, Martin Salö, Pernilla Stenström

**Affiliations:** ^1^Faculty of Medicine, Institution of Clinical Science, Lund University, 221 85 Lund, Sweden; ^2^Institution of Clinical Science, Lund University, Department of Pediatric Surgery, Skåne University Hospital, 221 85 Lund, Sweden

## Abstract

*Introduction*. In children treated surgically for first-time perianal abscesses, discovery and excision of concomitant fistulas may also be warranted.* Aim*. To evaluate children of varying age after incision and drainage of first-time perianal abscesses, examining recurrences rates with and without search for a fistula.* Method*. A retrospective review was conducted, analyzing children (ages 0–15 years) treated for first-time perianal abscesses at a tertiary pediatric surgical center, with a minimum follow-up of 6 months.* Results*. A total of 104 patients subjected to 112 treatments for first-time perianal abscesses were eligible. Surgical procedures constituted 84 (75%) of treatments, searching for fistulas in 49 (58%). In 34 (69%), fistulas were confirmed and treated. In the surgically treated subset, the recurrence rate was higher if no attempt was made to exclude a fistula (46%), as opposed to confirmed absence of a fistula (27%) or concurrent fistulotomy (9%; *p* = 0.02). Younger patients showed a higher recurrence rate (12/26; 46%), compared with older counterparts (11/58; 19%) (*p* = 0.002).* Conclusion*. In children surgically treated for first-time perianal abscess, recurrence rates appear to be lowered by locating and treating coexisting fistulas.

## 1. Introduction

Perianal abscess is an anorectal disorder affecting not only adults but also children [1] who may then experience considerable discomfort and possibly fever [2]. A strong male predominance is evident, with peak onset at ages <1 year [3, 4]. One theory is that such abscesses in children arise from abnormal crypts of Morgagni, resulting perhaps from excessive androgen stimulation or androgen-estrogen imbalance. These abnormal crypts are predisposed to infection and abscess formation [5–7]. Once the inflammatory focus surfaces at perianal skin, a fistula-in-ano may occur [1, 3, 5].

The most recommended and first-opted approach in this setting is conservative management. However, surgical intervention may be needed if patients become symptomatic, experiencing intense pain, fever, or diminished well-being. Surgical treatment consists of incision and drainage, done either alone or with search for a fistula. Fistulotomy may be warranted if a fistula is confirmed [7, 8].

Recurrences of perianal abscess and/or development of a fistula-in-ano after treatment of perianal abscess is reported at rates of 6–85% [1, 3, 6, 9, 10]. This wide range may be due to therapeutic differences (i.e., conservative management versus surgical intervention) or conventions in reporting and designating fistulas as recurrences or complications. Currently, there is no consensus on whether a child requiring surgical treatment of first-time perianal abscess should be checked for a fistula and possibly undergo fistulotomy, or whether this strategy applies only to instances of recurrent perianal abscesses or plainly visible fistulas [9, 11].

The primary objective of this study was to investigate recurrence rates of first-time perianal abscesses in children <15 years old, comparing outcomes in the aftermath of conservative management with those after surgical treatment, with or without search for a fistula.

## 2. Methods

### 2.1. Settings and Patients

All children enrolled in this study were treated at a tertiary center for specialized pediatric surgery, servicing children aged 0–15 years in an area of 1.8 million inhabitants. The children included were all referred from pediatricians or general practitioners for further assessment by a pediatric surgeon. A roster of six surgeons performed all operations, each conducting corresponding preoperative evaluations and postsurgical follow-up monitoring. Choice of treatment (conservative versus surgical) and type of surgical procedure were at the surgeon's discretion.

### 2.2. Study Design

Data for this retrospective study were gathered by reviewing medical records of all children admitted to the Department of Pediatric Surgery between January 2008 and April 2014. Patients treated for perianal abscesses were screened using the International Classification of Disease (ICD) Codes K61.0 and K60.3. Patients with systemic disorders, inflammatory bowel conditions, or Hirschsprung's disease were excluded from study, given their predisposition for such abscesses. Minimum follow-up time for patient participants was 6 months. Patient variables assessed included gender, age at onset of perianal abscess, surgeon-assessed abscess size and location, any antibiotic use, type of treatment, and recurrence status. Four therapeutic subsets were defined as follows: conservative treatment or incision and drainage with no search for a fistula, with no fistula found (upon search), or with fistulotomy (fistula confirmed). Treatment selection was at the surgeon's discretion. No imaging studies were included.

Patients were assigned to one of two groups (0–3 months and >3 months), based on patient age at time of treatment.

### 2.3. Definitions

Conservative treatment consisted of nonsurgical therapies, with or without use of antibiotics. Treatment with antibiotics was defined as any instance of antibiotic administration or prescription for perianal abscess, either as a conservative measure or in conjunction with surgery. Various antibiotics used alone or in combination were cefuroxime, metronidazole, and trimetoprim sulfa.

Abscess sizes were computed from reported diameters, each estimated by the operative surgeon and recorded in the medical chart. Abscess area was calculated as follows: *A* = *πd* ^2^/4.

The location of each abscess was mapped with the patient supine, dividing the perianal area into four quadrants (upper, lower, left, and right) relative to anus at center. Any abscess developing twice in the same quadrant was considered recurrent and was therefore excluded. On the other hand, any abscess developing anew in a different quadrant is qualified as a first-time abscess.

Surgical treatment consisted of incision with drain placement. All incisions were performed via monopolar diathermy or scalpel, followed by abscess debridement and drain placement (sutured to the skin edge). Drains were removed 2-3 days postoperatively. All surgical procedures were performed with patients under general anaesthesia.

A fistula was defined as a communication between mucosa and skin. In all instances, fistulas were confirmed by the use of a lacrimal probe to demonstrate a tract from anus to abscess, after incising the abscess. Searches not involving a lacrimal probe were regarded as “no search.” Fistulotomy was achieved through cautery, applying monopolar diathermy to the lacrimal probe. According to departmental protocol, fistulas were categorized as involving or not involving the sphincter.

### 2.4. Statistical Analysis

Fisher's two-tailed exact test was applied to analyze dichotomous variables, and Mann-Whitney *U* test was used for analysis of ranked results. The Freeman-Halton extension of Fisher's exact probability test was used for three- and four-row two-column analyses. All statistical calculations relied on standard software (SPSS v22.0.0.0 for Windows; SPSS Inc., Chicago, IL, USA), setting significance at *p* < 0.05. A statistician was responsible for all statistical analyses.

### 2.5. Ethical Considerations

The study was performed in accord with guidelines set forth in the Helsinki Declaration, and protocol approval was granted by the Regional Ethical Review Board (registration Number 2010/49). Data were coded prior to analytics execution, and results were presented in a manner that prohibited individual recognition.

## 3. Results

### 3.1. Patients and Treatments

**Figure 1:**
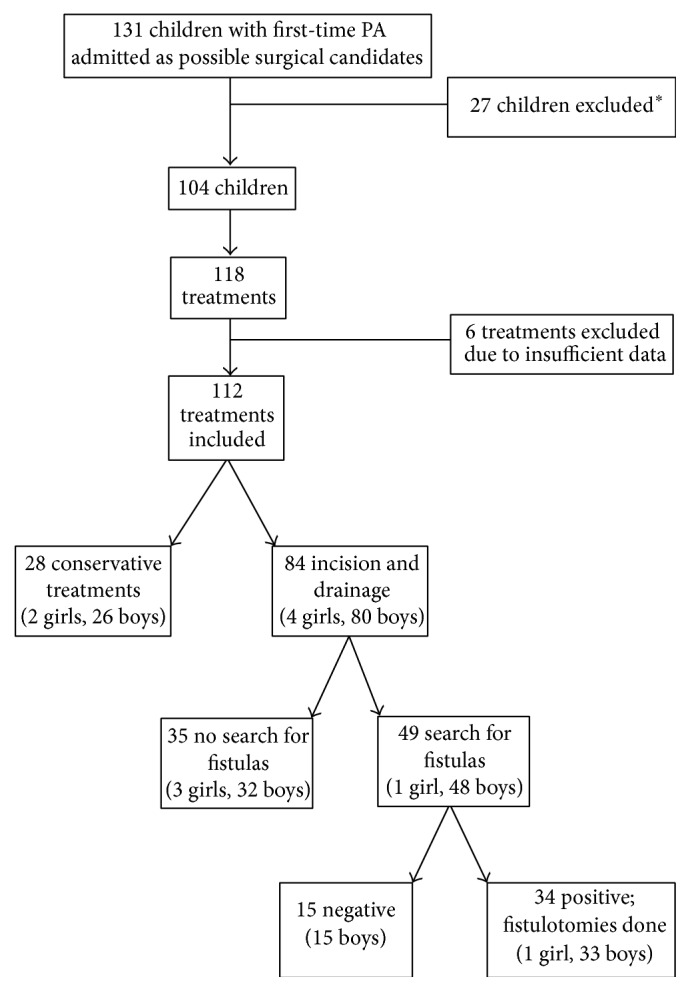
Flow chart of patients and treatments utilized for first-time perianal abscess (PA) in children 0–15 years old. ^*∗*^Exclusions: anorectal malformation (*n* = 1); Hirschsprung's disease (*n* = 1); Crohn's disease (*n* = 6); pilonidal abscess (*n* = 4); chronic anal fissure (*n* = 2); hemorrhoids (*n* = 2); no abscess found/disease inconclusive (*n* = 7); sarcoma (*n* = 1), intra-abdominal intestinal abscess (*n* = 1); needle incisions (*n* = 2).

A total of 131 patients admitted to the department with first-time perianal abscesses were identified initially as study candidates. Upon excluding 27 patients with underlying diseases, a total of 104 patients (male: 99; female: 5) were included in the final analysis, 11 of whom developed new abscesses in other quadrants (second location: 11; third location: 3). Thus, a total of 118 first-time perianal abscesses were treated. Because follow-up was not feasible in six instances, 112 fully documented treatments ultimately remained for final analysis (Figure 1). Median patient age at time of treatment was 5 months (range: 8 days–15 years). Median follow-up period was 3 years (range: 0.5–7 years).

### 3.2. Outcomes after Conservative Treatments

Of the 112 admissions for first-time perianal abscess, 28 (25%) involved conservative treatment with operations performed in 84 (75%) (Figure 1). Two of the six girls admitted were treated conservatively, whereas the other four (67%) underwent surgical procedures. Perianal abscesses managed conservatively were associated with a 25% (7/28) recurrence rate, which did not differ significantly from that of surgically treated abscesses (27%, 23/84; *p* = 1.0).

### 3.3. Abscess Size and Location

**Figure 2:**
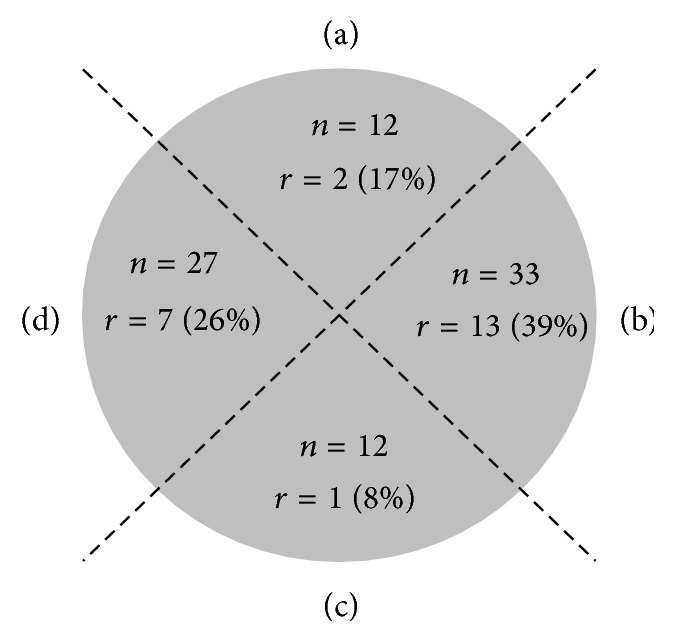
With patients supine, abscesses were mapped in four quadrants of perianal region, detailing locations in all 84 surgical interventions. Recurrence rates (*r*) did not differ significantly by quadrant (*p* = 0.171, Fisher-Freeman-Halton extension of Fisher's exact probability test). *n*: number of treatments; *r*: recurrences (%).

Abscess size was recorded in 38 (45%) surgical interventions and in 13 (46%) instances of conservative treatment. Median size (1.0 cm^2^; range: 0.2–4.0 cm^2^) of abscesses managed conservatively was significantly smaller, relative to surgically treated abscesses (3.5 cm^2^; range: 0.3–30.0 cm^2^; *p* = 0.001). Abscess locations were categorized in clinical descriptions as lateral to, above, or beneath anus, demonstrating a clear predilection for lateral quadrants (Figure 2). None of the fistulas involved the sphincter.

### 3.4. Outcomes of Various Surgical Interventions

In 49 (58%) of surgical procedures, search for a fistula was also conducted, with fistulas confirmed in 34 (69%) instances. All confirmed fistulas were located below dentate line and were treated by fistulotomy (Figure 1 and Table 1). Three of the four girls treated surgically underwent incision and drainage, without search for a fistula.

**Figure 3:**
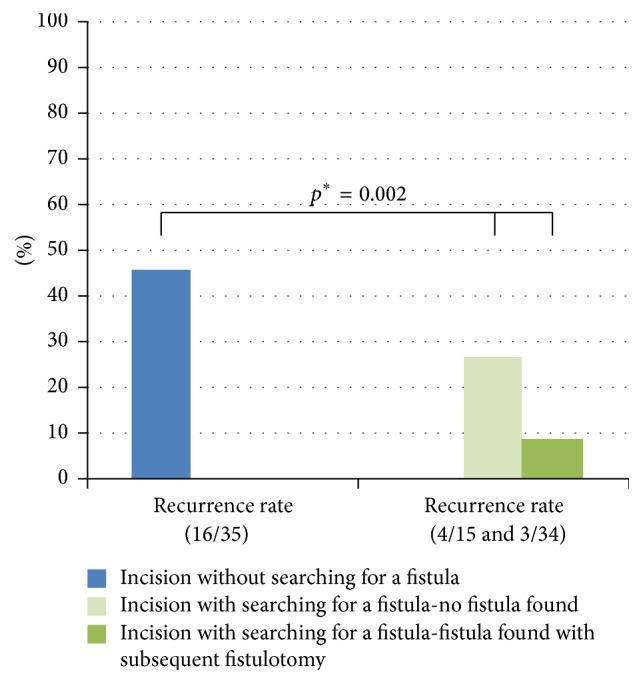
Recurrence rates for first-time perianal abscesses in children (median age: 0.5 years; range: 0–15 years) after incision and drainage, with and without searching for fistula-in-ano and concurrent fistulotomy. ^*∗*^Fisher-Freeman-Halton extension of Fisher's exact probability test.

Following surgical intervention, the recurrence rate was the lowest (9%) in procedures where search for a fistula was incorporated and a fistulotomy was performed concurrently. Overall, perianal abscesses that were surgically treated, with search for a fistula, resulted in fewer recurrences, compared with those omitting this search, regardless of whether or not a fistula was actually confirmed (Table 1, Figure 3). None of the four girls treated surgically experienced a recurrence, even without efforts to search for fistulas.

### 3.5. Outcomes after Various Surgical Interventions in Differing Age Groups

In 38 (34%) of all treatments, children were ≤3 months old. Thus, 66% of treatments were in children >3 months old. The distribution of surgical procedures, amounting to 26 (68%) in younger patients and 58 (78%) in the older age group, did not differ significantly (*p* = 0.259).

**Figure 4:**
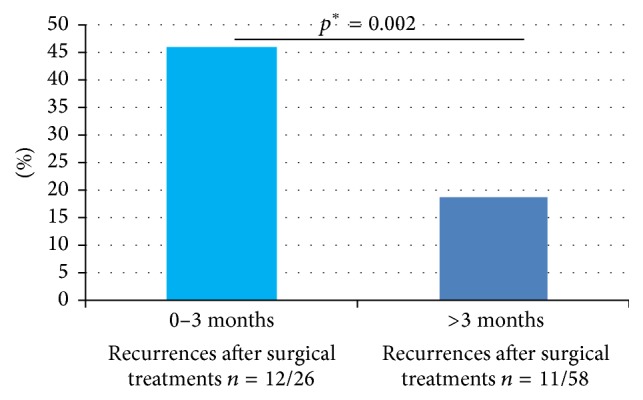
Recurrences after incision and drainage of first-time perianal abscess, stratified by age (0–3 months versus >3 months). ^*∗*^Fisher's exact test (two tailed).

Relative to older subjects (>3 months), where fistulas were searched for in 84% (49/58), significantly fewer young patients (38%; 10/26) were subjected to such probing (*p* = 0.017). Overall, recurrent perianal abscesses were significantly more common in the youngest of patients (0–3 months), compared with older children (Figure 4). Likewise, recurrence rates in younger and older age groups were 63% and 32%, respectively, if incision and drainage alone were elected. Regardless of age group, recurrence rates were lowest after incision and drainage, with search for a fistula and fistulotomy (Table 2).

### 3.6. Impact of Antibiotics

**Figure 5:**
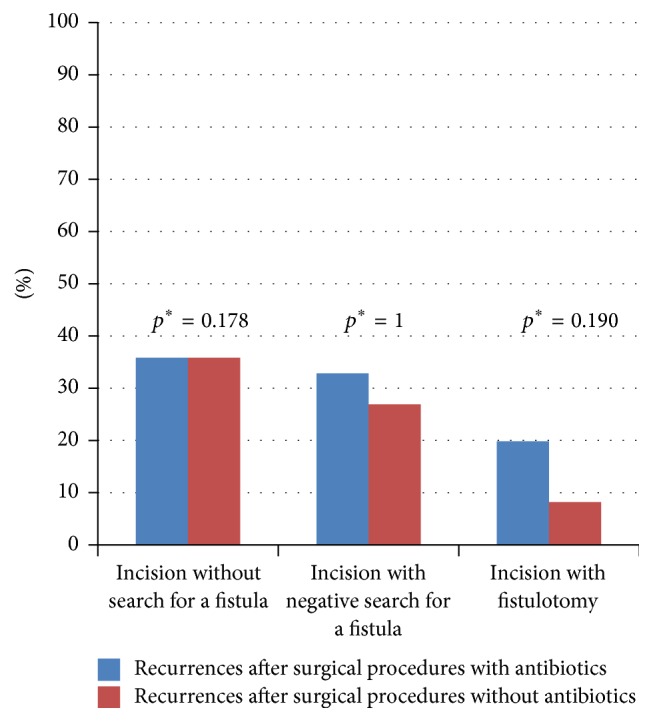
Comparison of first-time perianal abscess recurrences treated surgically, with and without antibiotics (recurrences shown by treatment subset). ^*∗*^Fisher's exact test (two tailed).

Antibiotics were used in conjunction with 29% (24/84) of surgical treatments. However, use or nonuse of antibiotics had no significant impact on the recurrence rate in patients undergoing incision and drainage; and a lower recurrence rate (not significant) was achieved by fistulotomy done without antibiotic use, as opposed to combining antibiotics with surgical procedures (Figure 5).

## 4. Discussion

If surgical treatment is needed for first-time perianal abscess in a child, our findings indicate that incision and drainage, with search for a fistula and concurrent fistulotomy, lower the risk of recurrence, compared with incision and drainage alone. Furthermore, first-time perianal abscesses in the youngest infants seemed less likely to recur if appropriately explored for a fistula and suitably treated. To our knowledge, no comparable therapeutic strategy for use in this setting has been published to date.

Treatments rendered in this study were predominantly surgical (75%), maybe because the children were referred to pediatric surgeons as presumptive surgical candidates, based on the severity of symptoms. In choosing treatment, surgeons were thus more inclined to actively intervene. However, recurrence rates did not differ significantly by the manner of treatment (conservative: 27%; surgical: 28%), which is aligned with previous reports and suggests that a conservative approach may be preferable, thus sparing children from general anaesthesia. In one publication, clinical courses of conservatively treated abscesses were followed in 18 infants <1 year old. Most (77%) of the subjects developed fistulas, all of which reportedly healed without surgical measures [7].

In our study, conservatively treated abscesses were smaller than those treated surgically. Size may well have influenced the surgeon's choice of treatment, but the reasons for surgical intervention were not studied, and there are no prior investigations addressing abscess size and treatment selection for comparison purposes. As such, no conclusion can be drawn to support the concept that larger abscesses are best treated by surgical intervention to avoid recurrences or development of fistulas. By dividing the perianal area into quadrants, abscess sites were monitored clinically as part of the study model. The results indicated that abscesses largely developed laterally to right or left of anus. This predilection may be attributable to the relatively softer nature of lateral tissues, which are thus more conducive to abscess formation.

In our cohort, an existing fistula was present in 69% of the patients examined, whereas previous reports of fistulas accompanying first-time abscesses or encountered during follow-up have cited rates of 77%–86% [4, 7, 12], with 54% in children <2 years old and 86% in older children [4]. However, examinations for fistulas during were not pursued in 42% of abscesses treated surgically during this study, so the actual rate presumptively is higher. The probing technique we utilized to search for fistulas, without first applying pressure* in situ* (via retractor) to an abscess before drainage, may also have contributed to our lower rate of discovery.

In the present study, only 9% of perianal abscesses subjected to fistulotomy recurred, which was 5-fold less than the recurrence rate following incision and drainage only. In line with this, a similar study of perianal abscesses (no first-time restriction) has reported 3-fold fewer recurrences in patients examined and treated for fistulas [13]. These data strongly support the contention that fistulas in perianal abscesses should be treated by fistulotomy. Because our study pertains to first-time perianal abscesses only, any general recommendation must await further validation. In any event, our data also suggest that a thorough search for fistulas during incision and drainage of first-time perianal abscesses may decrease the likelihood of recurrences in infants and children, even if no fistula is discovered. A vigorous search itself may therefore encourage better drainage and fewer recurrences. One argument against this approach may be the potential damage to anal sphincter during fistulotomy [14]. All fistulas in our cohort were situated without involving the sphincter and therefore carried no such risk. Unfortunately, preoperative imaging and follow-up monitoring of fecal incontinence were not conducted for verification.

When assessing outcomes of surgical procedures, one must also consider that many surgeons would not use a drain, choosing instead to widely deroof an abscess and pack with a biodegradable material. This less invasive technique was not standard within our department and therefore cannot be addressed. However, a future study comparing outcomes via differing techniques would be of interest.

As one observation, we noted that perianal abscesses recurred more frequently in younger (versus older) children. This age disparity is not corroborated by previous studies, which primarily involved older children [5, 11, 13]. One possible explanation is that we did not search as consistently for fistulas in the youngest subjects (0–3 months) as we did in older children, stemming perhaps from a desire to limit surgical trauma in infants and less expectation of detecting a fistula in the neonatal period.

According to our data, recurrence rates in perianal abscess treated surgically were not significantly impacted by the addition of antibiotics, although some earlier studies have reported fewer instances of fistulas if surgery and antibiotics are combined [3, 4, 8]. In our study, fistulotomy without antibiotics paradoxically resulted in a lower recurrence rate than that achieved by fistulotomy and antibiotics together. Subsets of patients within the group stratification (i.e., patients with severe versus milder disease) may be responsible. Our limited patient sampling does not permit conclusions in this regard, but the role of antibiotics in preventing recurrence is of environmental importance and merits further study.

A major weakness of this study was the selection bias imposed by a tertiary center, in that only patients preliminarily seen and treated by pediatricians or general practitioners were studied. Thus, related symptoms would likely have been more severe, and the tendency to refer very young infants is much greater. The extent of gender bias in surgical departmental admissions also was indeterminable without knowledge of initial presentations. The fact that <5% of study subjects were female clearly curtailed any analysis by gender. However, obvious bias did exist in selecting treatment, which was at the surgeon's discretion. The lack of an established protocol for treatment of perianal abscesses freely introduced bias through individual surgical implementation. Hence, our study outcomes will be difficult to compare with other investigations going forward.

Another limitation is the study's retrospective design, casting doubt on patient compliance during follow-up and validity of recurrence rates as a consequence. After surgically placed drains were removed, patients were instructed to contact our department at first sign of recurring or new abscess formation. It may be that, in spite of such instructions, recurrences were conservatively treated at nearby regional hospitals or health centers, without the surgeon's knowledge. Even at baseline, assurances that all abscesses were in fact first-time occurrences were hindered by difficulties in obtaining reliable and precise histories of previous perianal abscess/fistula treatments for patients admitted from other hospitals. The fact that we chose to register an abscess developing anew in a differing quadrant as a first-time occurrence may have created clustering issues in terms of unidentified fistulas, individual biologic predisposition for abscess development, or undiagnosed underlying disease.

The retrospective design may additionally have led to approximations and shortfalls in data on abscess size or location and antibiotic use; and our broad definition of antibiotic use may have influenced outcomes. A reliable subanalysis, comparing outcomes with single-dose perioperative antibiotics and prolonged courses of oral antibiotics, likewise was not possible in the absence of a standard therapeutic protocol.

It is conceivable that alternate stratification of patients by milk-fed status, body mass index, active infectious disease, or presence of diarrhea may have divulged other interesting outcome patterns relative to abscess recurrence or therapeutic options. More meticulous detailing of patient information in a larger prospective study would surely be beneficial for analysis of outcomes. We are now planning a prospective randomized study where pediatric candidates requiring surgical incision of first-time perianal abscesses must meet specific inclusion criteria to qualify. Subjects with associated fistulas would also be randomly assigned to either fistulotomy or conservative treatment.

Results of this study, however limited, will hopefully contribute to the development of general guidelines for managing children with first-time perianal abscesses, with more definitive answers provided through future investigations.

## 5. Conclusion

First-time pediatric perianal abscess requiring surgical intervention, incision, and drainage, with search for a fistula and concurrent fistulotomy (if indicated), is a seemingly preferential approach, carrying less risk of recurrence than incision and drainage alone.

## Figures and Tables

**Table 1:** Comparison of recurrences (frequency) after various treatments of first-time perianal abscesses in children. Median patient age: 0.5 years (range: 0–15 years).

	Treatment		Treated *n*	Recurrences *n* (%)	*p* value^*∗*^
Conservative treatment			28	7 (25)	1
Surgical incision and drainage			84	23 (27)	
	Without searching for a fistula		35	16 (46)	*0.003*
	With searching for a fistula		49	7 (14)	
		No fistula found	15	4 (27)	0.179
		Fistulotomy	34	3 (9)	

^*∗*^Fisher's exact probability test (two tailed).

**Table 2:** Distribution of various surgical treatments by age, comparing recurrence rates.

			0–3 months old		>3 months old		
	Treatment		Treated	Recurrences	Treated	Recurrences	*p* value^*∗*^
			*n*	*n* (%)	*n*	*n* (%)	
Surgical incision and drainage			26	12 (46)	58	11 (19)	*0.002*
	Without searching for a fistula		16	10 (63)	19	6 (32)	0.095
	With searching for a fistula		10	2 (20)	39	5 (13)	0.620
		Negative findings	3	1 (33)	12	3 (25)	
		Positive findings; fistulotomies done	7	1 (14)	27	2 (7)	

^*∗*^Fisher's exact test (two tailed).
